# Asymptomatic Polyvascular Abnormalities in Community (APAC) Study in China: Objectives, Design and Baseline Characteristics

**DOI:** 10.1371/journal.pone.0084685

**Published:** 2013-12-26

**Authors:** Yong Zhou, Yang Li, Liang Xu, Jie Xu, Anxing Wang, Xiang Gao, Shouling Wu, Wen Bin Wei, Xingquan Zhao, Jost B. Jonas

**Affiliations:** 1 Department of Neurology, BeijingTianTan Hospital, Capital Medical University, Beijing, China; 2 Beijing Tongren Eye Center, Beijing Tongren Hospital, Beijing Ophthalmology and Visual Science Key Laboratory, Capital Medical University, Beijing, China; 3 Beijing Institute of Ophthalmology, Beijing Tongren Hospital, Capital Medical University, Beijing, China; 4 Channing Laboratory, Department of Medicine, Brigham and Women’s Hospital, and Harvard Medical School, Boston, Massachusetts, United States of America; 5 Department of Nutrition, Harvard University School of Public Health, Boston, Massachusetts, United States of America; 6 Department of Cardiology, Kailuan Hospital, Hebei United University, Tangshan, China; 7 Department of Ophthalmology, Medical Faculty Mannheim of the Ruprecht- Karls-University, Heidelberg, Germany; Massachusetts General Hospital, United States of America

## Abstract

**Objective:**

The population-based “Asymptomatic Polyvascular Abnormalities in Community (APAC) Study was designed to examine prevalence and associations of asymptomatic polyvascular abnormalities (APA) in a general population. In this report, the objectives, design and baseline data of the APAC study are described.

**Methods:**

The study included 5,440 participants (40.1% women) with an age of 40+ years who were randomly selected from the population of the Kailuan Study which included 101,510 employees and retirees of the Kailuan Co. Ltd, a large coal mine industry located in Tangshan, Hebei, China. Exclusion criteria were previous cerebral stroke, transient ischemic attacks and coronary heart disease. In 2010 and 2011, information on potential cardiovascular risk factors was collected and all participants underwent transcranial Doppler sonography, measurement of the ankle brachial index, and bilateral carotid duplex sonography. In a first follow-up examination in 2012/2013, retinal photography and spectral-domain optical coherence tomography were additionally performed. In a planned long-term follow-up, data from clinical examinations and laboratory tests and the occurrence of cardiovascular or cerebrovascular events will be collected to build up a predicting model for the risk of ischemic events.

**Results:**

At baseline, mean age of the participants was 55.2±11.8 years, and men showed a significantly (*P*<0.001) higher prevalence of arterial hypertension (55.5% vs. 36.5%) and hyperlipidemia (50.7% vs. 46.0%) and a higher blood homocysteine concentration (18.68±10.28µmol/L versus 11.69±6.40µmol/L).

**Conclusions:**

The APAC is the first study to prospectively evaluate the relationship between intracranial arterial stenosis, retinal nerve fiber layer changes, retinal microvascular signs, and the eventual development of cerebrovascular or cardiovascular events.

## Introduction

Stroke is the leading cause of death in China and accounts for approximately 20% of all deaths causes [1]. Intracranial arterial stenosis (ICAS) of major arteries in the brain is one of the most common pathomechanisms for the development of an ischemic stroke [2-4]. In view of the potentially devastating consequences of an ischemic stroke, its prevention is of high importance. Prevention includes detecting patients at risk of eventually developing an ischemic attack [5]. Recent population-based studies have applied transcranial Doppler sonography (TCD) to diagnose an intracranial arterial stenosis [6-13]. Other studies have shown that retinal microvascular abnormalities such as focal arteriolar narrowing and arteriovenous nicking and retinopathy signs such as retinal hemorrhages and cotton-wool spots have prognostic importance to predict the eventual development of a cerebral stroke [14-17]. 

Recently, localized defects of the retinal nerve fiber layer (RNFLD), in addition to retinal microvascular abnormalities, have been described to be associated with different grades of arterial hypertension. In a recent study on 359 arterial hypertensive patients and 331 arterial normotensive individuals, the odds ratios for the association between localized RNFLDs and various grades of arterial hypertension were higher than the odds ratios for the association between the retinal microvascular abnormalities and the grades of arterial hypertension [18]. Other, hospital-based and population-based studies have revealed correlations between a diffuse thinning of the retinal nerve fiber layer (RNFL) and non-glaucomatous optic nerve damage the causes of which included cerebrovascular events [19-21]. The RNFL can be examined by simple ophthalmoscopy in daily practice and it can be measured by optical coherence tomography (OCT) which allows the detection of localized RNFLDs and the quantification of a diffuse thinning of the RNFDL [19,21].

In view of the relative easiness of the assessment of the RNFL, also by a non-ophthalmologist, and in view of that the association between the status of the intraocular RNFL as developmental part of the brain and the incidence of cerebrovascular events has not been explored yet, we designed the APAC to evaluate in a cross-sectional manner the relationship between the status of the RNFL and ICAS; and to examine longitudinally the correlation between the RNFL status and the incidence of cerebrovascular events. Additional goals are to examine in a cross-sectional manner and in a longitudinal manner the correlations between retinal microvascular abnormalities and the cerebrovascular status as assessed by TCD with particular emphasis on ICAS; and to compare the diagnostic and prognostic value of the RNFL status parameters with the diagnostic precision of the retinal microvascular abnormalities to forecast a cerebrovascular insult. The current report describes the design, rationale and baseline information of APAC.

## Methods

### Ethics Statement

The study was performed according to the guidelines of Helsinki Declaration and was approved by both the Ethics Committee of the Kailuan General Hospital, Beijing Tongren Hospital and Beijing Tiantan Hospital. The approval will be renewed every 5 years. Written informed consent was obtained from all participants. 

### Study Design and Population

**Figure 1:**
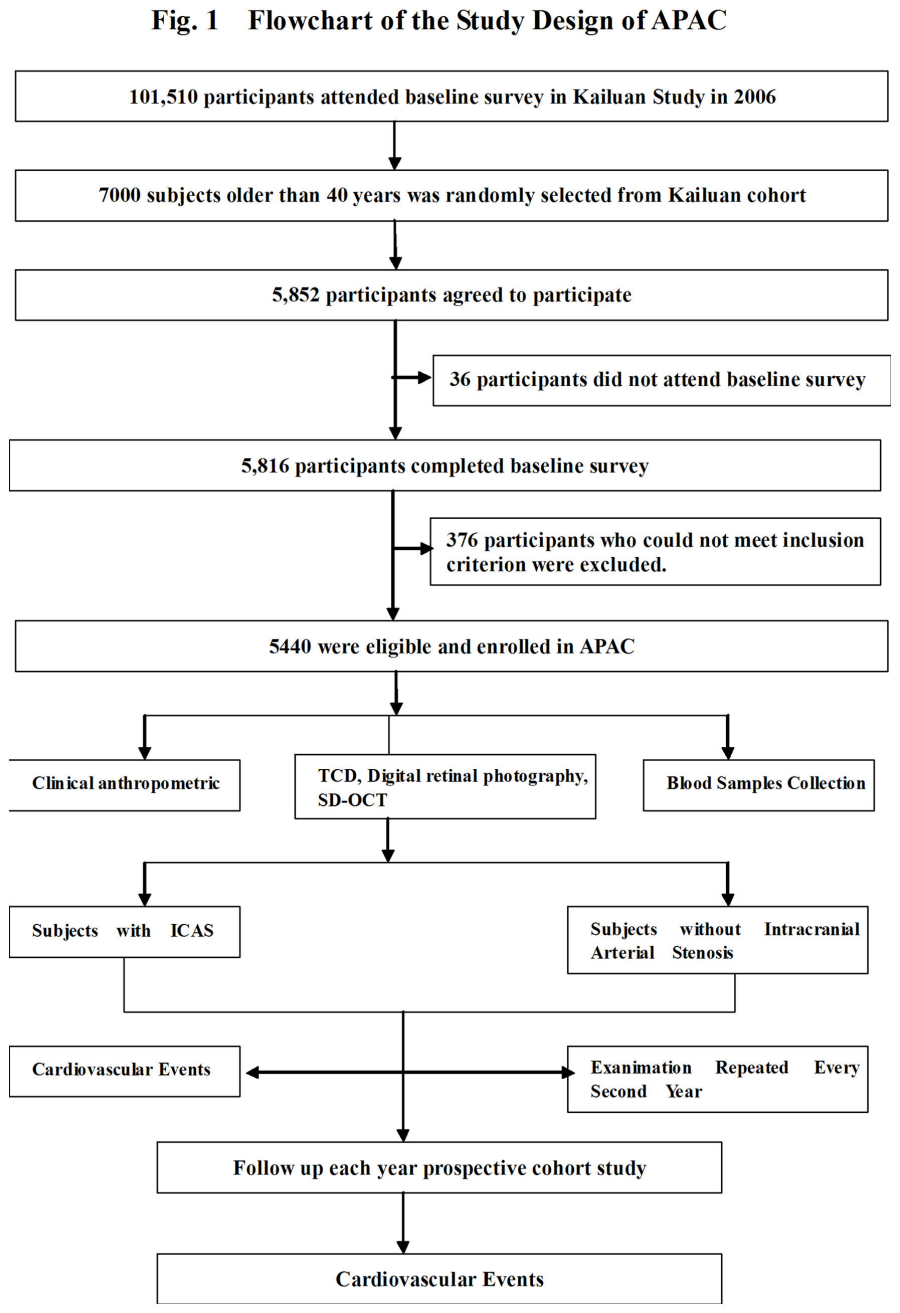
Flowchart of the Asymptomatic Polyvascular Abnormalities in Community (APAC) Study.

The Asymptomatic Polyvascular Abnormalities Community study (APAC) is a community-based, prospective, long-term follow-up observational study, to investigate the epidemiology of asymptomatic polyvascular abnormalities in Chinese adults. The study cohort was a sub-population of a previously described population of the Kailuan study which included a total of 101,510 employees and retirees (81,110 men) of the Kailuan (Group) Co. Ltd, a large coal mine industry located in Tangshan, Hebei Province (Figure 1) [22]. The city of Tangshan with approximately 7.2 million inhabitants in 2006 is situated 150 km southeast of Beijing and is a center of the coal mining industry. From June 2010 to June 2011, a sample of 7000 subjects older than 40 years was randomly selected from the Kailuan cohort, using a stratified random sampling method by age and gender based on the data of the Chinese National Census from 2010. The sample size was calculated based on detection of a 7% event rate with 0.7% precision and an α value of 0.05. The response rate was assumed to be >80%. A total of 5,852 subjects agreed to participate in the APAC study and 5,816 people eventually completed the baseline data collection. Among these 5,816 individuals, 376 subjects did not meet the following inclusion criteria (1) no history of stroke, transient ischemic attack, and coronary disease at baseline as assessed by a validated questionnaire; and (2) absence of neurologic deficits for stroke which was estimated by experienced doctors. Finally, a total of 5,440 participants were eligible and were included into APAC study. 

### Assessment of ICAS

Transcranial Doppler was performed by two experienced neurologists with portable examination devices (EME Companion, Nicolet, Madison, WI, USA). ICAS was diagnosed according to the peak flow velocity based on published criteria [7,19]. An arterial stenosis was defined by a peak systolic flow velocity of >140 cm/s for the middle cerebral artery, of >120 cm/s for the anterior cerebral artery, >100 cm/s for the posterior cerebral artery and vertebra-basilar artery, and >120 cm/s for the siphon internal carotid artery. In addition, the presence of turbulences or sound and the location of an abnormal blood flow velocity in a segment of a vessel or along the whole vessel were noted. In the absence of temporal windows, the examination was performed through the orbits to locate intracranial signals [23]. Subjects with poor insonation windows were considered as having no stenosis.

### Assessment of Retinal Photography

All study participants underwent fundus photography using a non-mydriatic digital fundus camera (fundus camera Type CR6-45NM; Canon, Ōta, Tokyo, Japan). Simultaneous stereoscopic 45° color fundus photographs centered on the optic disc (Diabetic Retinopathy Study standard field 1) and on the macula (Diabetic Retinopathy Study standard field 2) were taken for each eye. Using the fundus photographs and applying the protocol of the Atherosclerosis Risk in Communities study, we assessed the retinal vascular abnormalities of generalized retinal arteriolar narrowing, focal arteriolar narrowing, arteriovenous nicking and isolated retinopathy lesions (microaneurysms, cotton wool spots, retinal hemorrhages, or hard exudates) [24]. To assess generalized retinal vascular changes, the retinal vessel diameters were measured using computer assisted quantitative assessment software (IVAN; University of Wisconsin, Madison, WI), as described and applied in previous population-based studies [25]. Average retinal arteriolar or venular width was calculated by means of the Parr–Hubbard formula and was presented as the central retinal arteriolar equivalent or central retinal venular equivalent. The arteriovenous ratio was calculated as central retinal venular equivalent divided by the central retinal arteriolar equivalent. Generalized retinal arteriolar narrowing was defined as an arteriovenous ratio less than the lowest quartile of the arteriovenous ratio value in the normal control group [26]. The procedure has been described in detail elsewhere [27]. Arteriolar focal narrowing was considered to be present if the involved vessel had a diameter of at least 50 um or approximately one third the diameter of a major vein at the disc margin and if the narrowed part of the vessel had a caliber less than or equal to two-thirds of the caliber of the proximal and distal arterial segments. Arteriovenous nicking was present if there was a tapering or narrowing of the venous blood column on both sides of an arteriovenous crossing. Arteriovenous crossings located within half an optic disc diameter of the disc margin were excluded because, in that region, the vein usually crossed over the artery. Isolated retinopathy lesions (microaneurysms, cotton wool spots, retinal hemorrhages, and hard exudates) were graded as either present or absent. 

Presence of retinal emboli and presence of retinal vein occlusion was also assessed. Retinal emboli were intravascular lesions seen either along the course of retinal arterioles or at a bifurcation. They were graded as cholesterol (reflective or refractile), fibrin-platelet (dull) or calcific (chalky white) [28]. Recent central retinal vein occlusion was characterized by the presence of retinal edema, optic disc hyperemia or edema, scattered superficial or deep hemorrhages, and venous dilatation. Old retinal vein occlusions were characterized by occluded and sheathed retinal veins. Branch retinal vein occlusion involved a more localized area of the retina in the sector of the obstructed venule and was characterized by scattered superficial and deep retinal hemorrhages, venous dilatation, intraretinal microvascular abnormalities, and occluded and sheathed retinal venules. An ischemic type of vein occlusion was characterized by retinal neovascularization that was distinguished from intraretinal collateral vessel formation between an occluded and non-occluded region, in the case of branch retinal vein occlusions, or by very thin retinal vessels in the affected region [29]. 

### Assessment of Spectral-Domain Optical Coherence Tomography (SD-OCT)

SD-OCT images of the optic nerve head, macula and adjacent retina were obtained using a portable spectral-domain OCT system (iVue SD-OCT; Optovue Inc, Fremont, California, USA). The iVue SD-OCT used a superluminescent diode scan with a center wavelength of 840 ± 10 nm to provide high resolution images. A 6 x 6 mm^2^ raster scan was centered on the optic disc and macula. Each raster was composed of 101 B-scans each of which included 512 A-scans (acquisition time, 2.2 seconds). All OCT scans were obtained through undilated pupils. Quality assurance checks were carried out. Images with RNFL segmentation algorithm failures, motion artifacts, poor focusing or a scan score index <40 and images not centered were excluded from the assessment. Two experienced examiners (JL YL) scanned all study participants. Measurements of both eyes of each study participant were taken in a sitting position. For further analysis, we used the average RNFL thickness, the mean RNFL thickness in each quadrant, and the mean RNFL thickness in each of the twelve 30° clock-hour sectors. 

### Assessment of Extracranial Arterial Stenosis (ECAS)

Each participant underwent a bilateral carotid duplex ultrasound to evaluate for carotid stenosis as a part of standard diagnostic workup. Carotid stenosis (≥50%) was graded based on recommendations from The Society of Radiologists in Ultrasound Consensus Conference [30]. 

### Assessment of Peripheral Arterial Disease (PAD)

The ankle brachial index (ABI) measurement was calculated using a standard method [31]. Systolic blood pressure was measured with a handheld 5-MHz Bidirectional Doppler probe (Hokanson MD6 Doppler with MD6VR Chart Recorder; Bellevue, Wash). Pressures in each leg were determined and the ABI was calculated separately for each leg. An ABI <0.90 in either leg was considered as marker for the presence of a PAD, and an ABI ≥0.90 was considered normal. Elevated ABI values of ≥1.40 suggested poorly compressible leg arteries and were excluded from the statistical analysis. 

### Assessment of Demographic Variables and Cardiovascular Risk Factors

Information on demographic variables (e.g., age, gender, household income, level of education, and previous history of diseases) was collected through questionnaires. The participants were stratified by age into two categories: 40-59 years old and ≥60 years old. The average monthly income of every family member was reported as “<¥1,000” , “¥1,000-3,000” or “≥¥3,000”. The level of education was categorized as “illiteracy or primary school”, “middle school” or “high school or higher”. Information regarding previous history of diseases mainly contained arterial hypertension and dyslipdemia. Information on smoking, physical activity and dietary intake were collected by questionnaires. The smoking status was classified as “never”, “former”, or “current” according to self-reported information. Physical activity was classified as very active (exercise >80min/week), moderately active (exercise: 1~80min/week) or inactive (exercise: none). Salt intake was classified as low (salt: <6g/day), medium (salt: 6~10g/day) or high (salt: >10g/day).

Body weight (to the nearest 0.1 kg) and body height (to the nearest 0.1 cm) were measured, and the body mass index (BMI) was calculated as body weight (kg) divided by the square of height (m^2^). Blood pressure was determined to the nearest 2 mm Hg using a mercury sphygmomanometer with a cuff of appropriate size. Two readings of systolic blood pressure (SBP) and diastolic blood pressure (DBP) were taken at a five-minute interval. The average of the two readings was used for the current data analysis. If the two measurements differed by more than 5 mm Hg, an additional reading was taken, and the average of the three readings was used. BMI was divided into categories of <25 kg/m^2^ , 25 to 29.9 kg/m^2^, or ≥30 kg/m^2^. Arterial hypertension was defined based on the following information alone or in combination: 1) as presence of a history of arterial hypertension; 2) using antihypertensive medication; or 3) a systolic blood pressure ≥140 mm Hg, or a diastolic blood pressure of ≥90 mm Hg. Diabetes mellitus was defined as a self-reported history, current treatment with insulin or oral hypoglycemic agents, or fasting blood glucose level ≥126 mg/dl. Dyslipidemia was defined by a self-reported history, current use of cholesterol lowering medicine, or a total cholesterol level ≥220 mg/dl or triglyceride ≥150 mg/dl or low density lipoprotein ≥160 mg/dl.

### Genetic and Biomarker Data Collection

At each examination Blood samples were collected from the antecubital vein in the morning under fasting conditions. They were stored in vacuum tubes containing EDTA (Ethylene DiamineTetraacetic Acid) for storage. Fasting blood glucose was measured with the hexokinase/ glucose-6-phosphate dehydrogenase method. Cholesterol and triglyceride concentrations were measured enzymatically (inter-assay coefficient of variation: < 10% (Mind Bioengineering Co. Ltd, Shanghai, China)). Blood samples were also measured using an auto-analyzer (Hitachi 747; Hitachi, Tokyo, Japan) at the central laboratory of the Kailuan General Hospital. For all participants, serum creatinine, cholesterol, high-density lipoproteins (HDL-C), low-density lipoproteins (LDL-C), triglycerides and glucose levels were assessed. In subgroup analysis studies, various biomarkers of blood cells, serum and plasma were measured including the C-reactive protein, homocysteine, steroids (e.g. estrogens, androgens, vitamin D, Lp-PLA2), insulin and glycosylated hemoglobin HbA1c. 

### Follow-Up and Outcome Assessment

The study participants will be followed up by face-to-face interviews once every two years in a routine medical examination up to December 31, 2015, or up to the occurrence of a final event as defined in the study, or occurrence of death. The follow-ups are performed by physicians and nurses who are masked to the baseline data. The outcome information for subjects not participating in the follow-up examination is obtained by checking death certificates from provincial vital statistics offices, discharge summaries from the 11 hospitals, and medical records from the medical insurance. 

The primary outcome is the first occurrence of stroke, either the first non-fatal stroke event or death by stroke. A non-fatal stroke is defined as a focal neurological deficit of vascular origin and of sudden onset and which lasts >24 hours. Stroke is diagnosed according to the World Health Organization (WHO) criteria combined with brain computed tomography (CT) or magnetic resonance (MR) confirmation, and classified into three main types: brain infarction, intracerebral hemorrhage, and subarachnoid hemorrhage [32]. The criteria are consistent across all participating hospitals. 

All stroke records are reviewed by two independent stroke specialists. If the two specialists disagree, the event adjudication committee reviews the case and makes the final decision. All stroke outcomes are checked by the Data Safety Monitoring Board and Arbitration Committee for Clinical Outcomes. 

The first occurrence of coronary heart disease is an additional outcome event. Incident coronary heart disease is defined as the occurrence of a fatal or non-fatal myocardial infarction (I21), other forms of acute (I24) or chronic ischemic (I25) heart disease, sudden (cardiac) death (I46 and R96), death caused by ventricular fibrillation (I49), or death resulting from congestive heart failure (I50) during follow-up [33]. Other outcomes include heart failure and atrial fibrillation [34,35]. 

### Data Management and Statistical Analyses

The data management system including the statistical analysis is performed using the SAS software (version 9.1; SAS Institute, Cary, North Carolina, USA) or the SPSS software (SPSS for Windows, version 21.0, IBM-SPSS, Chicago, IL). As first step, the distributions (mean ± standard deviation) of the parameters are calculated. The normal distribution of the parameters is assessed using the Kolmogorov-Smirnoff test. As second step, the parameters are compared between the groups, using either the student’s t test for paired or non-paired samples of normally distributed parameters or the Wilcoxon-Mann-Whitney test or the Wilcoxon test for non-parametric variables. The Chi-squared test is applied for the comparison of categorical variables. As third step of the analysis, multivariate associations between the various parameters will be performed using multivariable regression analyses or binary regression analyses. As fourth step, a longitudinal assessment of associations and a survival analysis for the combination of major vascular events and for each vascular event will be performed using the Kaplan-Meier curves in relation to the presence/absence of intracranial stenosis, retinal microvascular abnormalities, retinopathy signs and RNFL defects. Cox multivariate regression models will be used to compare the probability of having a vascular event in the follow-up cohorts, adjusting for the necessary covariates. The relative risk (hazards ratios) will be presented with their corresponding 95% confidence intervals. The level for statistical significance will be set at α = 0.05 (two -tailed).

## Results

A total of 5440 subjects (2183 (40.1%) women) were enrolled in the APAC study. The basic characteristics of the study population including age, level of education, income level and previous disease history were described and compared. Women were younger and had a higher level of education compared to men (*P*<0. 001), while men showed a higher prevalence of arterial hypertension (55.5% versus 36.5%) and hyperlipidemia (50.7% versus 46.0) (Table 1). 

**Table 1:** Baseline Demographic Characteristics (Mean ± Standard Deviation) of the Asymptomatic Polyvascular Abnormalities in Community (APAC) Study Stratified by Gender.

	Total	Women	Men	*P*-Value
n	5440	2183	3257	
Age (Years)	55.17 ± 11.79	52.3 ± 9.73	57.1 ± 12.62	<0.001
Education (n, %)				
Illiteracy / Primary School	662 (12.2)	185 (8.5)	477 (14.6)	<0.001
Middle School	2394 (44)	878 (40.2)	1516 (46.6)	
High School or Higher	2383 (43.8)	1120 (51.3)	1263 (38.8)	
Income (n, %)*				
<¥1,000	1153 (21.2)	462 (21.2)	691 (21.2)	<0.001
¥1,000-3,000	3595 (66.1)	1570 (71.9)	2025 (62.2)	
≥¥3,000	690 (12.7)	151 (6.9)	539 (16.6)	
Previous History of Disease				
Arterial Hypertension (n, %)	2604 (47.9)	796 (36.5)	1808 (55.5)	<0.001
Diabetes mellitus (n, %)	655 (12)	216 (9.9)	439 (13.5)	
Dyslipidemia (n, %)	2655 (48.8)	1005 (46.0)	1650 (50.7)	

*P*-Value: Statistical significance of difference between men and women

The prevalence of risk factors for asymptomatic polyvascular abnormalities (APA) such as smoking, BMI, physical activity, salt intake and the homocysteine level differed significantly between men and women, while both gender did not vary significantly in high-sensitive C-reactive protein concentration (Table 2). Specifically, smoking was significantly more prevalent in men than women (31.9% versus 1.8%; *P*<0.01). BMI index was higher in women than in men for the age group of <25 years, whereas it was higher in men than women for the age group of 25 to 30 years. There was no significant difference between gender in BMI for the age groups of >30 years (Table 2). Homocysteine levels were categorized into 4 quartiles as shown in Table 2. Men as compared with women were significantly found more often in the higher 3^rd^ and 4^th^ quartiles, and consequently had significantly higher mean values 18.68 ± 10.28 µmol/L versus 11.69 ± 6.4 µmol/L) (Table 2). 

**Table 2:** Distribution of Risk Factors in APAC Stratified by Gender.

	Total (n, %)	Women (n, %)	Men (n, %)	*P*-Value
Smoking				
Never	3400 (62.5)	2137 (97.9)	1263 (38.8)	<0.01
Former	303 (5.6)	7 (0.3)	296 (9.1)	
Current	1737 (31.9)	39 (1.8)	1698 (52.1)	
Body Mass Index (kg/m^2^)				
<25	2907 (53.4)	1251 (57.3)	1656 (50.8)	<0.01
25 to 29.9	2178 (40.0)	783 (35.9)	1395 (42.8)	
≥30	355 (6.5)	149 (6.8)	206 (6.3)	
Physical Activity				
Very Active	2178 (40.0)	827 (37.9)	1351 (41.5)	<0.01
Moderately Active	1388 (25.5)	658 (30.1)	730 (22.4)	
Inactive	1874 (34.4)	698 (32.0)	1176 (36.1)	
Salt intake				
Low	1135 (20.9)	535 (24.5)	600 (18.4)	<0.01
Medium	3242 (59.6)	1309 (60.0)	1933 (59.3)	
High	1063 (19.5)	339 (15.5)	724 (22.2)	
High-Sensitive C-Reactive Protein (mg/L, median)				
1st tile(<0.5)	1277 (23.5)	487 (22.3)	790 (24.3)	0.03
2st tile(0.5^~^1.0)	1442 (26.5)	623 (28.5)	819 (25.1)	
3st tile(1.0^~^2.2)	1379 (25.3)	534 (24.5)	845 (25.9)	
4st tile(>2.2)	1342/ (24.7)	539 (24.7)	803 (24.7)	
Homocysteine (µmol/L)				
1st tile(<9.6)	1376 (25.3)	910 (41.7)	466 (14.3)	<0.01
2st tile(9.6~13.6)	1344 (24.7)	635 (29.1)	709 (21.8)	
3st tile(13.6~19.3)	1357 (24.9)	415 (19.0)	942 (28.9)	
4st tile(>19.3)	1363 (25.1)	223 (10.2)	1140 (35.0)	
Mean ± Standard Deviation	15.87 ± 9.56	11.69 ± 6.40	18.68 ± 10.28	<0.01

*P*-Value: Statistical significance of difference between men and women

## Discussion

More than 7 million Chinese currently suffer from the sequelae of stroke, with another 2 million subjects newly diagnosed and 1.4 million Chinese affected by a recurrence of stroke each year [1]. Intracranial arterial stenosis of the major arteries is the most common factor leading to ischemic stroke worldwide. Intracranial arterial stenosis is not an isolated disease, but it is related to generalized atherosclerosis affecting other body regions such as the carotid artery, the coronary artery or peripheral arteries [36,37]. Table 3 compares previous studies on intracranial stenosis [38-43]. Classic vascular risk parameters have now been combined with new markers as carotid intima-media thickness, ankle-arm index or high sensitive C-reactive protein values to assess the individual vascular risk. Prior to the occurrence of a stroke, most patients may already have pathological changes in their intracranial arteries, even though these changes can be asymptomatic. 

**Table 3:** General Characteristics of Studies on Asymptomatic Polyvascular Abnormalities.

First Author	Sample Size	Design	Prevalence (%)
Wong KS (2007)[7]	590 Chinese population aged ≥40 years	Cross-sectional, population-based	6.9 (ICAS)
Wong KS (2007) [8]	3,057 Chinese patients with at least one vascular risk factor	Cross-sectional, hospital-based	12.6 (MCA stenosis)
Huang HW (2007) [6]	1,068 Chinese subjects aged ≥ 50 years	Cross-sectional, pop.-based	5.9 (MCA stenosis )
Uehara T(2005) [49]	425 Japanese asymptomatic patients	Cross-sectional, hospital-based	3.5 (ICAS)
Park KY(2006) [50]	835 Korean adults	Cross-sectional, hospital-based	3.0 (ICAS)
Elmore EM (2003) [51]	510 asymptomatic persons	Cross-sectional ,hospital-based	12.9 (ICAS)
Lopez-Cancio (2012) [52]	933 stroke-free Caucasians	Cross-sect. and cohort study, popul.-based	8.6 (ICAS)
Elizabeth Selvin (2004) [53]	2174 participants aged 40 years and older	Cross-sectional, population-based	4.3 (PAD)
Marjoleinde Weerd (2010) [54]	23,706 participants	population-based studies	0 to 3.1 (ECAS)
APAC study (2012)	5,440 stroke-free Chinese	Ongoing cohort study, population-based	13.2 (ICAS); 6.7 (ECAS) 7.8 (PAD)

Similarities between the retinal and cerebral small vessels suggest that examination of the relationship between retinal microvascular abnormalities and stroke and its subtypes may help understanding the etiology of stroke and risk for stroke. Data from various large population-based cohorts have provided new insights into the prognostic value of retinal microvascular signs. The Atherosclerosis Risk in Communities (ARIC) was a prospective US population-based community study of 15,792 individuals aged 45–64 years with 86% of the cohort having retinal photography performed at the third follow-up [17]. The Blue Mountains Eye Study was an Australian population-based cohort study including participants 49 years of age or older [28]. The Rotterdam Scan Study was a Dutch population-based cohort study in which 6780 subjects participated in the ophthalmic part of the study [16]. The US-based Cardiovascular Health study assessed retinal photographs of about 4000 persons [44]. The Beaver Dam Eye Study in Wisconsin examined retinal vascular signs in persons 43–84 years of age [45]. These studies demonstrated that retinal microvascular signs were associated not only with stroke risk factors (e.g. arterial hypertension) [46,47] and history of vascular disease [48], but were also independently related to a variety of other vascular risk factors [49] and important outcomes such as incident stroke [17,22], subclinical cerebral infarct [24], white matter abnormalities on magnetic resonance imaging (MRI) [16] and cognitive impairment [50]. Other studies of patients with symptomatic atherosclerotic disease (recent ischemic stroke, myocardial infarction, or peripheral arterial disease) demonstrated that retinal arteriolar narrowing as defined from retinal photographs was related to the presence of white matter lesions and lacunar infarcts detected on MRI [51]. Other researchers reported that retinal microvascular blood flow was reduced in persons with white matter lesions and lacunar infarction [52], and that the histopathology of retinal arterioles and cerebral arterioles was similar in patients with stroke [53]. Retinal vessels and cerebral vessels in general share similar anatomic and physiologic characteristics and changes [54]. Retinal examination may thus be a surrogate of cerebral vessel examination and the retina could be considered as a ‘window’ to the cerebral vessels.

Spectral-domain OCT (SD-OCT) permits high-resolution retinal imaging with resolution of the separate retinal layers and thuds allows measurement of the thickness of the retinal nerve fiber layer and the retinal ganglion cell complex. Both layers forming the first part of the optic nerve have not been evaluated by SD-OCT to predict the incidence of stroke in a large epidemiological study. 

Since as in any study of a design such as the one of the APAC, problems may occur with the data collection, in using the various instruments and in performing the different procedures, additional steps will be incorporated into the study design. All examiners involved with measuring the study parameters will undergo a standardized central training and certification, and in regular intervals of about 6 months, a re-certification should follow. This certification process will include all interviewers and health professionals responsible for the clinical and laboratory examinations to ensure that the procedures are performed according to the study protocol. In addition, the inter-observer reproducibility and intra-observer reproducibility shall be tested by mixing 10 copies of each of 20 images under the whole volume of samples undergoing the routine assessment procedure. These 20 images with their copies will be re-assessed in a masked manner. In general the measured data will be collected in electronic datafiles which will daily be copied into a safety datafile. 

Potential limitation of our study should be discussed. First, the study is based on a randomly selected subgroup of the participants of the large Kailuan Study which includes employees and retirees of the Kailuan Company. One may argue that despite its large study sample, the Kailuan Study population may not be representative for the population of the city of Tangshan or the province of Hebei. The large sample size of the study allows however forming subgroups of actual coal miners, retired coal miners, and office employees. The study design may therefore enable to examine and compare the study parameters within a coal miner specific group, to compare the influence of previous working and lifestyle conditions with the influence of actual working and lifestyle conditions (among the retired coal miners), and to assess the study parameters in an unspecific non-coal mining population group (office workers and other employees). In addition, the study population was selected using a stratified random sampling method by age and gender based on the data of the Chinese National Census from 2010. Second, a magnetic resonance imaging of the brain to detect clinically silent strokes will not routinely be performed. Strength of the study is its size and the stability and low mobility of the study population what may facilitate longtime follow-up examinations. 

In conclusion, our report describes the rationale, study hypothesis and methodology of the Asymptomatic Polyvascular Abnormalities in Community (APAC) Study. In 2010 and 2011, information on potential cardiovascular risk factors was collected and all participants underwent transcranial Doppler sonography, measurement of the ankle brachial index, and bilateral carotid duplex ultrasound. In a first follow-up examination in 2012/2013, retinal photography and spectral-domain optical coherence tomography were additionally performed. In a planned long-term follow-up, data from clinical examinations and laboratory tests and the occurrence of cardiovascular or cerebrovascular events will be collected to build up a predicting model for the risk of ischemic events. The APAC is the first study to prospectively evaluate the relationship between intracranial arterial stenosis, retinal microvascular signs, retinal nerve fiber layer changes, and the eventual development of cerebrovascular or cardiovascular events. 
