# Correction: Asymptomatic Polyvascular Abnormalities in Community (APAC) Study in China: Objectives, Design and Baseline Characteristics

**DOI:** 10.1371/annotation/0b52d557-24d9-4b49-a5a4-75f32b117afc

**Published:** 2014-01-10

**Authors:** Yong Zhou, Yang Li, Liang Xu, Jie Xu, Anxin Wang, Xiang Gao, Shouling Wu, Wen Bin Wei, Xingquan Zhao, Jost B. Jonas

There was an error in the spelling of the fifth author's name. The correct spelling is Anxin Wang.

